# Identification of hub genes associated with decreased fertility in male mice of advanced paternal age

**DOI:** 10.3389/fcell.2025.1520387

**Published:** 2025-04-04

**Authors:** Yang Gao, Ting Zhang, Yan Wang, Haitao Lv, Xiangming Yan, Longlong Fu, Ying Liu

**Affiliations:** ^1^ Institute of Pediatric Research, Children’s Hospital of Soochow University, Suzhou, Jiangsu, China; ^2^ Department of Pediatrics, The First People’s Hospital of Lianyungang, Xuzhou Medical University Affiliated Hospital of Lianyungang (Lianyungang Clinical College of Nanjing Medical University), Lianyungang, China; ^3^ Department of Urology, Children’s Hospital of Soochow University, Suzhou, Jiangsu, China; ^4^ Department of Cardiology, Children’s Hospital of Soochow University, Suzhou, Jiangsu, China; ^5^ Reproductive Health Research Centre/Human Sperm Bank,NHC Key Laboratory of Frontiers and Technologies in Reproductive Health, National Research Institute for Family Planning, Beijing, China

**Keywords:** hub genes, decreased fertility, male, mice, advanced paternal age

## Abstract

**Introduction:**

Aging and delayed parenthood are major social concerns. Men older than 35 years, which is an advanced paternal age, experience reduced sperm quality and fertility.

**Methods:**

In this study, 12-month-old mice served as a model for males of advanced paternal age. RNA sequencing (RNA-seq) of epididymides from 2- and 12-month-old mice was performed.

**Results:**

Spermatogonia and sperm counts were significantly lower in these mice. We identified 449 differentially expressed genes by RNA-seq. Altered pathways were enriched using Gene Ontology and Kyoto Encyclopedia of Genes and Genomes (KEGG) analyses. Moreover, nine hub genes were identified from the DEGs, along with DEGs associated with mitochondria.

**Discussion:**

These results could enhance understanding of the molecular mechanisms underlying decreased male fertility in men of advanced paternal age and may aid in developing targeted treatment for male infertility related to aging.

## 1 Introduction

Aging and delayed parenthood are major societal concerns (Amaral et al., 2013; Pohl et al., 2021). Male reproductive function declines with age, and men older than 35 years—considered to be of advanced paternal age (Soğukpınar et al., 2024)—often experience reduced sperm quality and fertility (Harris et al., 2011).

Sperm quality and male fertility are closely related with spermatogenesis, which begins in the seminiferous tubules of the testes. Spermatogonial stem cells in the basal lamina of the seminiferous tubules differentiate into spermatocytes, which subsequently undergo meiosis to form round spermatids. Sperm develop from these round spermatids through morphological changes and the removal of excess cytoplasm (Zhu et al., 2023a; Zhu et al., 2023b; Wei et al., 2020). Although fully developed in the testes, sperm are not yet mature; they acquire motility and fertilization capacity in the epididymis (Chen et al., 2021). The epididymis is composed of three regions: the caput, corpus, and cauda. Sperm maturation occurs in the caput and corpus, with functionally mature sperm stored in the cauda (Shum et al., 2009). Therefore, analyzing sperm in the epididymis is suitable for investigating functional changes associated with advanced paternal age.

Previous studies have used mice aged 21 or 24 months as aging models to investigate the relationship between aging and declining male fertility using RNA sequencing or single-cell RNA sequencing of the epididymal tissue (Zhuang et al., 2023; Endo et al., 2024). According to the murine lifespan information provided by the Jackson Laboratory (https://www.jax.org/), mice aged of this age are roughly equivalent to human males in their 60 s. However, this study focuses on men of advanced paternal age (older than 35 years) who may still desire biological children. Therefore, 12-month-old male mice, corresponding to human males in their 40 s, were used as an advanced paternal age model. RNA was extracted from the whole epididymis, and RNA-seq was performed. The RNA-seq data were analyzed to identify hub genes with altered expression in mice of advanced paternal age.

## 2 Materials and methods

### 2.1 Animals

All C57BL/6 strain mice were born and raised in standard experimental cages under controlled conditions (temperature = 25°C ± 2°C; humidity = 50 ± 5%). All mice were euthanized by CO2 asphyxiation. All animal experiments adhered to the National Institutes of Health Guide for the Care and Use of Laboratory Animals and were approved by the Animal Care and Use Committee of the Soochow University (SUDA20220906A01).

### 2.2 Histological examination

Testes were collected from 2- and 12-month-old male mice and fixed in 4% paraformaldehyde (Solarbio Science and Technology, Beijing, China) overnight. The testes were then embedded in paraffin and sectioned at 3-μm thickness for hematoxylin and eosin staining (Solarbio Science and Technology). Sections were scanned using a Pannoramic DESK (3D HISTECH, Budapest, Hungary) and imaged with a Pannoramic Scanner (C.V.2.4, 3D HISTECH).

### 2.3 Sperm motility and concentration analysis

The left epididymis of each mouse was dissected to obtain the sperm, following our previous methods (Liu et al., 2023). After CO2 asphyxiation, the left epididymis was quickly transferred into a tube containing 1 mL of 37°C Biggers–Whitten–Whittingham (BWW) solution and incubated for 15 min. Next, 10 μL of sperm solution was added into 2 mL of 37°C BWW solution in a 6-well plate, and six random fields from each sample were recorded using MShot Image Analysis System version 1.2.11.10. The percentage of motile sperm and sperm concentration in the recordings were calculated by professional technicians in a single-blind manner.

### 2.4 RNA-seq and bioinformatic analysis

Total RNA was extracted from the right epididymis of each mouse using TRIZOL (Life Science) and a DNA-free kit (Ambion). RNA-seq was conducted as previously described (Li et al., 2022).

### 2.5 Identification of differentially expressed genes (DEGs) and mitochondria-related DEGs (MitoDEGs)

All bioinformatics analyses were conducted in R software (version 4.2.1). Principal component analysis (PCA) was performed, and DEGs between the two groups were identified using the “limma” package, with |logFC| > 1 and Adjusted p-Value <0.05 as thresholds. The results were visualized using the “ggplot2” package. The MitoCarta3.0 database (https://personal.broadinstitute.org/scalvo/MitoCarta3.0/mouse.mitocarta3.0.html) containing 1,140 mouse mitochondria-related genes (MRGs), was used to intersect MRGs with DEGs, yielding MitoDEGs.

### 2.6 Functional enrichment analysis of DEGs

Gene Ontology (GO) analysis, encompassing molecular function (MF), biological processes (BP), and cellular components (CC), is a robust strategy for large-scale functional enrichment. The Kyoto Encyclopedia of Genes and Genomes (KEGG) contains extensive information on genomes, diseases, biological pathways, and medicines. To perform GO and KEGG enrichment studies, we used the “clusterProfiler” package. A statistically significant difference was determined at a threshold of *P* < 0.05, and the GO and KEGG results are visually represented.

### 2.7 Protein-protein interaction (PPI) network construction and hub gene identification

The PPI network was analyzed using the Search Tool for the Retrieval of Interacting Genes (STRING) database (http://string-db.org/) (version 12.0). To assess the potential PPI associations, genes obtained from previous analyses were mapped onto the STRING database. PPI pairs with a total value higher than 0.4 were selected. The resulting PPI network was visualized using Cytoscape software (version 3.9.1). Degree value of each node was calculated with the “Analyze Network” function in the software, and nodes with degree value ≥5 were selected for visualization. Hub genes were identified using three different methods—maximum clique centrality (MCC), degree, and neighborhood component centrality (MNC)—in the cytoHubba plug-in, with the results intersected to obtain the final hub genes.

### 2.8 Friendship analysis

Friendship analysis methods assess functional correlations between different genes within a pathway, suggesting that a gene is more likely to express if it interacts with other genes in the same pathway. These methods are widely used to identify core genes. To determine core genes among the hub genes, Symbol gene IDs were first converted to Entrez gene IDs using the “org.Mm.eg.db” package. GO entry annotation was performed; molecules not annotated to a GO entry were excluded from subsequent analysis. Functional correlations between hub genes were calculated using the “GOSemSim” package.

### 2.9 Statistical analysis

Sperm motility and concentration data were normally distributed, as indicated by the Shapiro–Wilk test. These data were analyzed using t-test in SPSS 20. All statistical bioinformatics analyses were performed using R (https://www.r-project.org/, 4.2.1 version).

## 3 Results

### 3.1 Abnormal structure of seminiferous tubule and decreased sperm concentration in 12-month-old mice

**FIGURE 1:**
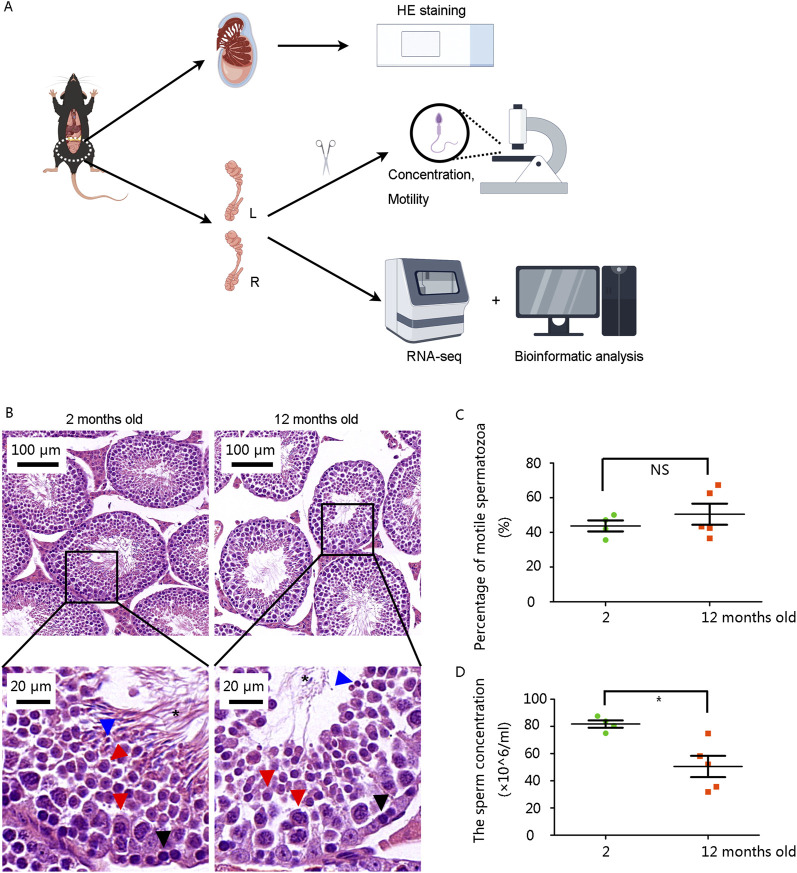
Structural abnormalities in seminiferous tubules and reduced sperm concentration in 12-month-old male mice **(A)** Schematic diagram of the study. **(B)** Hematoxylin and eosin staining results of testes. Scale bars are shown in the figures. Black arrows: spermatogonium, Red arrows: spermatocyte, Blue arrows: spermatid, asterisk: sperm. **(C)** The percentage of motile spermatozoa showed no significant difference. **(D)** Sperm concentration was significantly decreased in 12-month-old mice. *P < 0.05. Data were analyzed using Student’s t-test and presented as mean ± SEM.

The mice were raised for 12 months to model advanced paternal age, while mice aged 2 months served as the normal control (NC) group. Testes and epididymides were collected for subsequent examinations (Figure 1A). Multiple layers of germ cell types, including spermatogonia, spermatocytes, spermatids, and sperm, were observed in the seminiferous tubules of the testes in the 2-month-old mouse. The number of spermatogonia and sperm in the 12-month-old mice was significantly lower (Figure 1B).

Then we analyzed sperm parameters from left epididymis. Although the percentage of motile spermatozoa was not significantly altered (Figure 1C), sperm concentration was markedly decreased in the 12-month-old group (Figure 1D).

### 3.2 Identification and analysis of DEGs by RNA-seq of right epididymides

**FIGURE 2:**
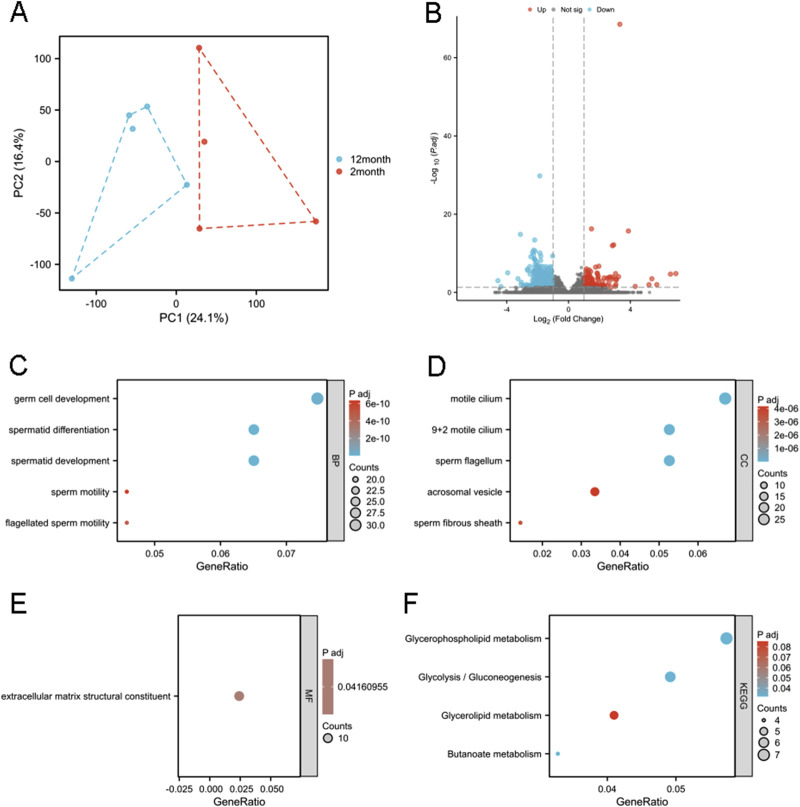
Functional enrichment analysis of the DEGs. **(A)** Principal component analysis of the RNA-seq results. PC1 and PC2 denote the first and second principal components explaining the maximum variance in the dataset (cumulative variance: XX%), visualizing the overall differences among samples. **(B)** A total of 449 DEGs identified, as shown in the volcano diagram. GO analysis including **(C)** BP, **(D)** CC, and **(E)** MF of the DEGs. **(F)** KEGG enrichment analysis of DEGs. Gene ratio represents the proportion of genes associated with a specific functional category among all differentially expressed genes (e.g., 10/100 indicates 10%), reflecting the enrichment level. Adjusted p-values (P adj) were calculated via multiple hypothesis testing correction to control false discovery rate, with P adj <0.05 considered significant.

RNA sequencing was performed on right epididymides from both the 2- and 12-month-old groups. Principal component analysis was conducted on the RNA-seq data (Figure 2A). Differential gene expression analysis was performed to identify potential genes associated with age-related decline in male fertility. Using an adjusted p-value threshold of 0.05, and | logFC | > 1.0, we identified a 449 DEGs, including 123 upregulated and 326 downregulated genes, presented in a volcano diagram (Figure 2B). GO and KEGG enrichment analyses were conducted to characterize DEGs and understand their biological activities. DEGs were classified into three functional groups: BP, MF, and CC. For BP term, DEGs were enriched in germ cell development, spermatid differentiation, spermatid development, sperm motility, and flagellated sperm motility (Figure 2C). CC terms for DEGs included cornified motile cilia, 9 + 2 motile cilia, sperm flagella, acrosomal vesicles, and sperm fibrous sheaths (Figure 2D). MF terms for DEGs were predominantly related to extracellular matrix structural components (Figure 2E). KEGG enrichment analysis showed that DEGs were primarily associated with pathways related to glycerophospholipids, glycolysis/gluconeogenesis, glycerolipids, and butanoate metabolism (Figure 2F).

### 3.3 PPI network construction and hub gene identification

**FIGURE 3:**
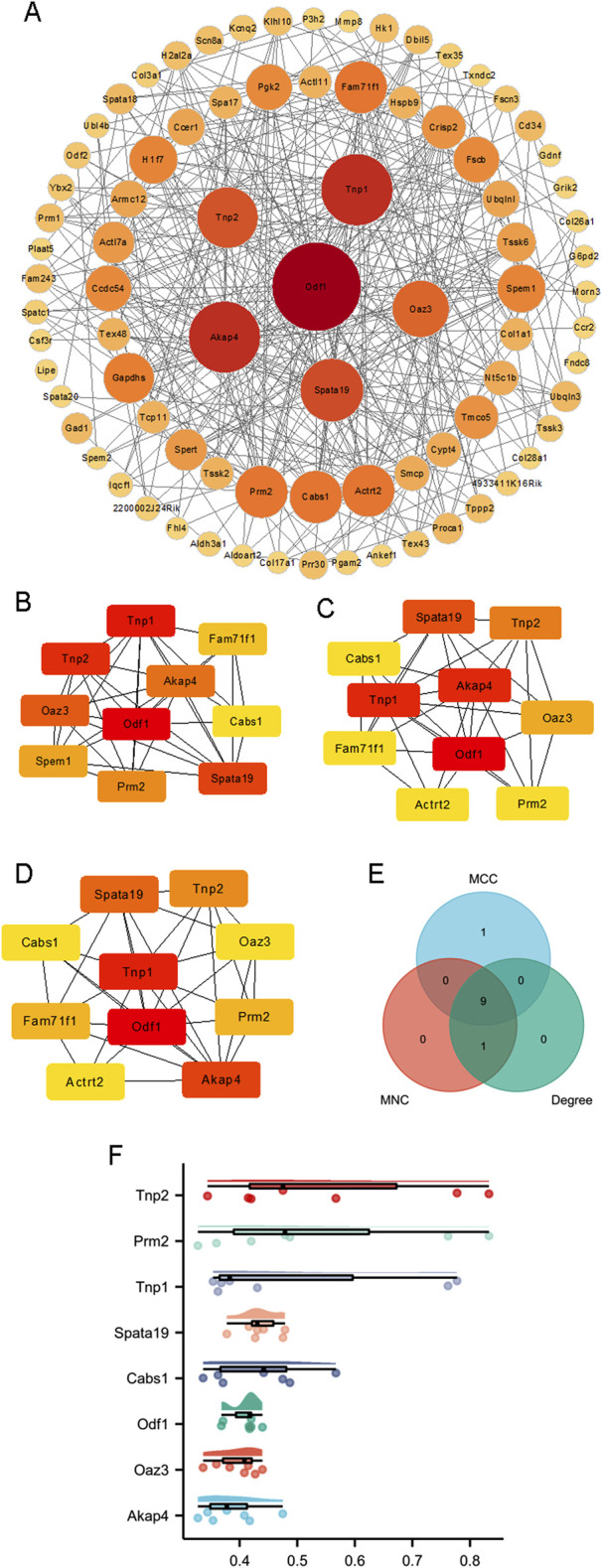
Construction of PPI networks. **(A)** The PPI networks of 83 proteins. Hub genes were calculated by **(B)** MCC, **(C)** degree, and **(D)** MNC. **(E)** Nine genes were identified by Venn analysis of the hub genes calculated by MCC, degree, and MNC. **(F)** Friendship analysis of the resulting hub genes. X-axis: Semantic similarity between genes. Y-axis: Genes sorted in descending order by mean similarity. Top genes exhibit the highest similarity and are identified as key genes.

PPI networks were constructed based on 83 proteins, as outlined in the Methods section (Figure 3A). Hub genes were identified using three distinct methods: MCC, degree, and MNC (Figures 3B–D). The hub genes calculated by each method were then intersected, and finally nine hub genes were obtained (Figure 3E), which were transition protein 2 (*Tnp2*), family with sequence similarity 71, member F1 (*Fam71f1*), protamine 2 (*Prm2)*, transition protein 1 (*Tnp1*), spermatogenesis associated 19 (*Spata19*), calcium binding protein (*Cabs1*), outer dense fiber of sperm tails 1 *(Odf1*), ornithine decarboxylase antizyme 3 (*Oaz3*), and A kinase anchor protein 4 (*Akap4*) (Table 1). A friend analysis of the hub genes showed that eight of these genes were included in the analysis (Figure 3F).

**TABLE 1:** The information of the hub DEGs^a^.

Genes	Subcellular location	Function
Tnp2 and Tnp1	Nucleus	Plays a key role in replacing histones for protamines in the elongating mammalian spermatids
Prm2	Nucleus	replacing histones for protamines in sperm during spermatogenesis
Spata19	Mitochondrion	Important for sperm motility and male fertility
Cabs1	Cytoplasm and Mitochondrion	Essential for maintaining the integrity of sperm flagella structure
Odf1	Cytoplasm	A component of the outer dense fibers of spermatozoa, and helps the activity of sperm tail
Oaz3	Nucleus and cytoplasm	Probably regulating the concentration of polyamines in haploid spermatids
Akap4	Cytoplasm	Major component of sperm fibrous sheath, which is essential for sperm motility

^a^ The information was obtained from Uniprot (www.uniprot.org). Last visit time was 8 August 2024.

### 3.4 DEGs associated with mitochondria

**FIGURE 4:**
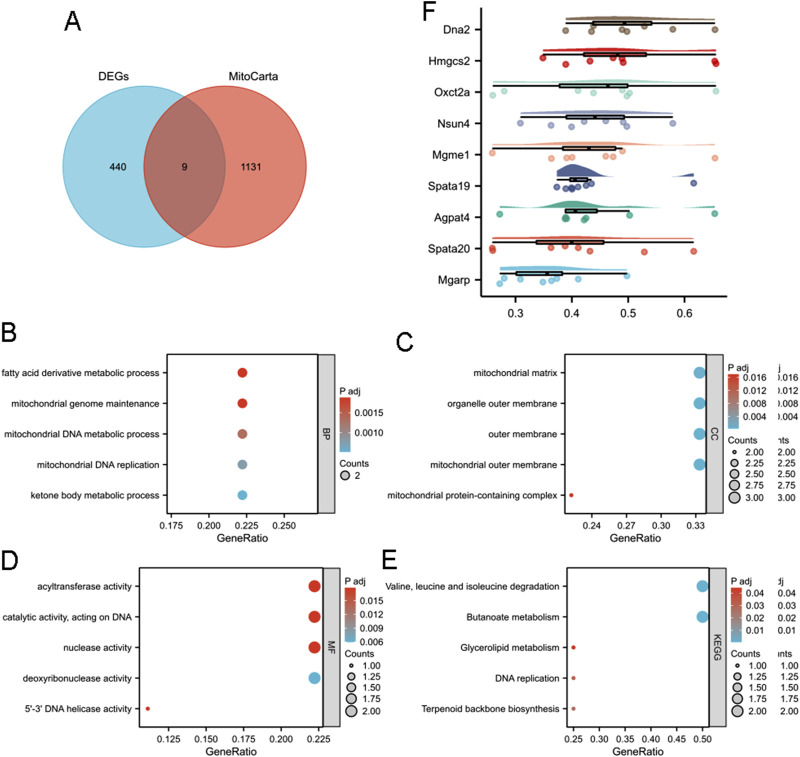
Functional enrichment analysis of mitochondria-associated DEGs. **(A)** Venn analysis of DEGs and MitoCarta data. GO analysis including **(B)** BP, **(C)** CC, and **(D)** MF of intersected MRGs and DEGs. **(E)** KEGG enrichment analysis of intersected MRGs and DEGs. **(F)** Friendship analysis of the resulting hub genes. X-axis: Semantic similarity between genes. Y-axis: Genes sorted in descending order by mean similarity. Top genes exhibit the highest similarity and are identified as key genes.

As mitochondria are essential organelles in sperm (Fu et al., 2024), we investigated their potential role in reduced male fertility associated with aging. Nine MitoDEGs (MitoCarta) were obtained from the intersection of the MRGs and DEGs (Figure 4A). These genes included DNA replication helicase/nuclease 2 (*Dna2*), 3-hydroxy-3-methylglutaryl-Coenzyme A synthase 2 (*Hmgcs2*), three-oxoacid CoA transferase 2A (*Oxct2a*), NOL1/NOP2/Sun domain family member 4 (*Nsun4*), mitochondrial genome maintenance exonuclease 1 (*Mgme1*), *Spata19*, 1-acylglycerol-3-phosphate O-acyltransferase 4 (*Agpat4*), spermatogenesis associated 20 (*Spata20*), and mitochondria-localized glutamic acid rich-protein (*Mgarp*).

GO and KEGG analyses were conducted on MitoDEGs to gain further insight into their biological functions. MitoDEGs were grouped into BP, MF, and CC terms. For BP terms, MitoDEGs were enriched in fatty acid derivative metabolic processes, mitochondrial genome maintenance, mitochondrial DNA metabolic processes, mitochondrial DNA replication, and ketone body metabolic processes (Figure 4B). CC terms for MitoDEGs included the mitochondrial matrix, organelle outer membrane, outer membrane, mitochondrial outer membrane, and mitochondrial protein-containing complexes (Figure 4C). MF terms for MitoDEGs primarily involved acyltransferase activity, catalytic activity acting on DNA, nuclease activity, deoxyribonuclease activity, and 5′-3′ DNA helicase activity (Figure 4D). Signal pathway analysis indicated that DEGs were enriched in pathways related to valine, leucine, and isoleucine degradation; butanoate metabolism; glycerolipid metabolism; DNA replication; and terpenoid backbone biosynthesis (Figure 4E). Friend analysis of MitoDEGs also suggested that *Spata19* is a key hub gene, potentially playing a critical role in mitochondrial function and contributing to reduced male fertility (Figure 4F).

## 4 Discussion

This study demonstrated decreased male fertility in 12-month-old mice compared to 2-month-old mice and explored the hub genes associated with this decline in an advanced paternal age mouse model.

Mitochondria are essential organelles in sperm, providing ATP for motility and acrosome reactions, both critical for male fertility (Fu et al., 2024). Mitochondrial dysfunction can disrupt sperm function (Wang et al., 2024; Pi et al., 2024). In this study, *Spata19* was identified as a potential key gene associated with decreased male fertility in advanced paternal age through multiple analyses (Figures 3A–F, 4A,F). *Spata19* is localized to the mitochondria (Mi et al., 2015) and regulates the architecture of the sperm midpiece architecture (Chen et al., 2022), indicating its involvement in spermatogenesis and sperm maturation (Rahimi Kalateh Shah Mohammad et al., 2020). *Spata19* has also been identified as an antigen in benign prostatic hyperplasia and prostate cancer (Wong et al., 2017). Previous research has shown that the antispermatogenic activity of nanoliposomes loaded with H. persicum phenolic compounds in BALB/c mice decreased the expression of *Spata19* in testicular tissues (Alami et al., 2023). Conversely, expression levels of *Spata19* may be useful in assessing the potential fertility of crossbred bulls (Saraf et al., 2022). These findings, along with the results of the present study, highlight the involvement of *Spata19* in male fertility across various models, further emphasizing its potential role in age-related male infertility.

GO analysis of the DEGs (Figure 2C) indicated alterations in (flagellated) sperm motility, potentially due to structural changes in flagellum and fibrous sheath (Figure 2D) and abnormalities in ATP production via glycolysis (Figure 2F), a major energy source for sperm (Tian et al., 2024). Additionally, enrichment of extracellular matrix structural components in MF terms (Figure 2E) suggests that the epididymal environment in the 12-month-old group may have changed, impacting sperm maturation and activity. Glycerophospholipids and glycerolipids are essential components of sperm membranes (Farrokhi et al., 2023; Garcia-Fabiani et al., 2017), and butanoate may be the target metabolite of damaged sperm DNA (He et al., 2024). Metabolism alterations (Figures 2F, 4B,C,E) may thus contribute to decrease male fertility through biochemical mechanisms.

RNA-seq has been performed on testes and cauda epididymides from 2- and 24-month-old mice (Endo et al., 2024). In 24-month-old (aged) testicular and epididymal tissues, the top 10 enriched biological process GO terms for upregulated genes were predominantly associated with inflammatory responses (e.g., “Inflammatory response”, “Neutrophil migration”) (Endo et al., 2024). Notably, key marker genes for cellular senescence (such as Cxcr5 and Il1a) spermatogenesis and germ cell development—including those governing undifferentiated spermatogonia (e.g., Dmrt1 and Lin28a), spermatogonial differentiation (e.g., Dazl and Sall4), meiosis (e.g., Dmc1 and Sycp1), and spermiogenesis (e.g., Adam2 and Adam3)—were not significantly downregulated in 24-month-old tissues. Similarly, marker genes for Sertoli cells (e.g., Ar, Wt1) and Leydig cells (e.g., Cyp17a1, Cyp11a1) showed no age-related downregulation (Endo et al., 2024). To dissect age-related cellular alterations in the male reproductive tract, single-cell RNA sequencing (scRNA-seq) was performed on the epididymal initial segment (EIS) of 3-month-old and 21-month-old (aged) mice (Zhuang et al., 2023). This analysis aimed to characterize cell-type composition and transcriptional landscape in the EIS during aging, with a focus on structural degeneration and immune-related changes. In our work, functional enrichment of DEGs in 12-month-old mice did not recapitulate the immune-related pathways that identified in prior studies. This discrepancy likely arises from our younger aging model (12 months vs 21 and 24 months), as immune senescence is age-dependent and progressive. For example, studies on the immune system of turquoise killifish demonstrated dramatic cellular and systemic changes with age, including increased inflammation, reduced antibody diversity, altered gut microbiota, and extensive DNA damage in immune progenitor cell clusters (Morabito et al., 2024). This findings suggests that, while 21- or 24-month-old mice may serve as a useful aging model, they may be less suitable for studying male fertility or advanced fertility specifically.

While our study identified key hub genes and pathways associated with age-related male fertility decline in mice, it is essential to contextualize these findings within the framework of human genetic and epigenetic heterogeneity. Genetic variability across populations, such as single nucleotide polymorphisms (SNPs) or copy number variations (CNVs) in hub genes, may alter their expression or functional roles in spermatogenesis and sperm maturation.For example, age-related hypermethylation of promoter regions of genes such as Dna2 and TNP2 has been associated with decreased sperm quality in older men (Phillips et al., 2019; Zheng et al., 2020).

Importantly, our interpretation of the RNA-seq data must take into account the genetic homogeneity of the C57BL/6 mouse strain used. While this model minimizes the interference of genetic variation, human populations exhibit great genetic and epigenetic diversity. In addition, lifestyle and environmental factors (e.g., diet, stress, toxin exposure) can induce epigenetic changes that interact with genetic predisposition to further complicate translational extrapolation (Soubry et al., 2013). Future studies combining multi-ethnic human cohorts or genetically diverse mouse models (e.g., collaborative cross-strains) could elucidate how genetic and epigenetic heterogeneity affects the molecular pathways identified herein. These approaches will improve the generalizability of our findings and inform personalized interventions for male infertility associated with aging.

Recent advancements in artificial intelligence (AI) have revolutionized the study of complex biological systems, including aging and reproductive biology. Machine learning (ML) and deep learning (DL) algorithms are increasingly employed to analyze high-throughput omics data, such as RNA sequencing, to identify subtle gene expression patterns and predict functional interactions among genes (Kulmanov and Hoehndorf, 2020). Furthermore, AI-powered platforms enable the integration of multi-omics data (e.g., genomics, proteomics, metabolomics) to uncover novel pathways linking mitochondrial dysfunction, metabolic alterations, and spermatogenic decline in aging males. Network-based AI models, such as graph neural networks (GNNs), can prioritize candidate genes for therapeutic targeting by analyzing protein-protein interaction (PPI) networks and gene co-expression patterns (Su et al., 2020). Future studies could leverage AI to predict fertility decline risk in men of advanced paternal age or optimize drug discovery targeting hub genes like Spata19.

In summary, RNA-seq analysis of epididymides from 12-month-old mice identified hub genes associated with decreased male fertility due to advanced paternal age. Bioinformatics methods were used to identify key pathways. Our findings provide insights into the molecular mechanism underlying reduced male fertility in men of advanced paternal age and may support the development of targeted treatment for age-related male infertility.

## Data Availability

The data can be obtained from the corresponding authors upon reasonable request.
